# Using genomic DNA-based probe-selection to improve the sensitivity of high-density oligonucleotide arrays when applied to heterologous species

**DOI:** 10.1186/1746-4811-1-10

**Published:** 2005-11-09

**Authors:** John P Hammond, Martin R Broadley, David J Craigon, Janet Higgins, Zoe F Emmerson, Henrik J Townsend, Philip J White, Sean T May

**Affiliations:** 1 Nottingham Arabidopsis Stock Centre, School of Biosciences, University of Nottingham, Sutton Bonington Campus, Loughborough, LE12 5RD, UK; 2 Plant Sciences Division, School of Biosciences, University of Nottingham, Sutton Bonington Campus, Loughborough, LE12 5RD, UK; 3 Warwick HRI, University of Warwick, Wellesbourne, Warwick, CV35 9EF, UK

**Keywords:** *Arabidopsis thaliana*, *Brassica oleracea*, Brassicaceae, cross-species, microarray, oligonucleotide, phosphate, phosphorus, plant nutrition, probe masking, transcriptomics

## Abstract

High-density oligonucleotide (oligo) arrays are a powerful tool for transcript profiling. Arrays based on GeneChip^® ^technology are amongst the most widely used, although GeneChip^® ^arrays are currently available for only a small number of plant and animal species. Thus, we have developed a method to improve the sensitivity of high-density oligonucleotide arrays when applied to heterologous species and tested the method by analysing the transcriptome of *Brassica oleracea *L., a species for which no GeneChip^® ^array is available, using a GeneChip^® ^array designed for *Arabidopsis thaliana *(L.) Heynh. Genomic DNA from *B. oleracea *was labelled and hybridised to the ATH1-121501 GeneChip^® ^array. *Arabidopsis thaliana *probe-pairs that hybridised to the *B. oleracea *genomic DNA on the basis of the perfect-match (PM) probe signal were then selected for subsequent *B. oleracea *transcriptome analysis using a .cel file parser script to generate probe mask files. The transcriptional response of *B. oleracea *to a mineral nutrient (phosphorus; P) stress was quantified using probe mask files generated for a wide range of gDNA hybridisation intensity thresholds. An example probe mask file generated with a gDNA hybridisation intensity threshold of 400 removed > 68 % of the available PM probes from the analysis but retained >96 % of available *A. thaliana *probe-sets. Ninety-nine of these genes were then identified as significantly regulated under P stress in *B. oleracea*, including the homologues of P stress responsive genes in *A. thaliana*. Increasing the gDNA hybridisation intensity thresholds up to 500 for probe-selection increased the sensitivity of the GeneChip^® ^array to detect regulation of gene expression in *B. oleracea *under P stress by up to 13-fold. Our open-source software to create probe mask files is freely available  and may be used to facilitate transcriptomic analyses of a wide range of plant and animal species in the absence of custom arrays.

## Introduction

High-density oligonucleotide (oligo) arrays are a powerful and widely used tool for large-scale gene-expression profiling [1]. One extensively validated oligo array type is available as commercial GeneChip^® ^technology (Affymetrix, Santa Clara, USA). GeneChip^® ^arrays use probe-sets rather than single oligos per gene, comprising of between 11 and 20 probe-pairs to quantify abundance for each transcript. Each probe-pair consists of a perfect-match (PM) and a mismatch (MM) probe. The PM probe is a 25-base sequence complementary to the target transcript, whilst the MM probe is identical to the PM probe with the exception of a single mismatch at the 13^th ^base. Transcript abundance can be calculated either from extrapolated hybridisation differences between PM and MM probes across a probe-set, or simply by using the PM data depending on the analysis used. GeneChip^® ^arrays provide reproducible, accurate data at high throughput rates which can be easily stored and compared across experiments [2-4]. An example of the wide-scale adoption of GeneChip^® ^technology can be seen within the plant sciences research community, where data from thousands of GeneChip^® ^arrays under large numbers of experimental challenges on the model plant *Arabidopsis thaliana *(L.) Heynh. are publicly-available [4]. Unfortunately, at present, GeneChip^® ^arrays are available for only a few species of eukaryotes. Thus, in contrast to *A. thaliana*, the transcriptomes of most agriculturally- or ecologically-important plant species are less extensively studied, since extensive sequence information and the fabrication of expensive custom arrays would be required before experimentation can begin.

One solution to this problem is to use GeneChip^® ^arrays designed for model organisms to study closely related species [5-12]. Whilst this strategy is feasible, studies conducted to date have not accounted for the probability that inefficient hybridisation of transcripts from the target species to GeneChip^® ^probes designed to the model species will attenuate the overall signal calculated across a probe-set [13]. For example, Ji et al. [13] describe a scenario where expression data analysed in Microarray Analysis Suite (MAS Version 5.0; Affymetrix), is based on the calculation that the weight carried by a probe within a probe-set is inversely related to its distance from the mean value of all probes within the probe-set. Thus, probes not generating signals due to sequence polymorphisms with the target organism will reduce the quality of information available from experiments using GeneChip^® ^arrays designed for a model species to monitor the transcriptome of a closely related species. Ji *et al. *[13] thus adopted an RNA-based probe-selection system, to study non-human mammalian transcriptomes with human GeneChip^® ^arrays, using probe mask files to exclude probes which hybridised weakly to their target transcript. However, this technique reduced the number of probe-sets available for transcriptome analysis by biasing the analysis towards the measurement of abundant transcripts.

To address the potential problems of using GeneChip^® ^arrays designed for one species to monitor the transcriptome of a closely related species, we have developed a novel technique to improve the sensitivity of high-density oligonucleotide arrays when applied to heterologous species. In contrast to Ji *et al. *[13], this technique is based on selecting probe-pairs based on the hybridisation efficiency of the PM oligonucleotide probe with genomic DNA from a target species for which the GeneChip^® ^was not originally designed. We initially used the *A. thaliana *ATH1-121501 GeneChip^® ^array to study the transcriptome of *Brassica oleracea *L., which represents several important crops, including cabbage, kale, broccoli, Brussels sprout, cauliflower and kohlrabi, and half of the *B. napus *(oil-seed rape) genome. *Brassica oleracea *was chosen because the ancestral lineage of *Arabidopsis *and *Brassica *(family Brassicaceae) diverged only 12 to 19 million years ago [14]. The effects of genomic DNA-based probe-selection on estimates of transcriptional regulation in *B. oleracea *were quantified under an imposed physiological stress of phosphorus (P) deprivation. Probe mask files increased sensitivity of the ATH1-121501 GeneChip^® ^array when detecting regulation of gene expression in *B. oleracea *under P stress by up to 13-fold. The response of *B. oleracea *to P stress was compared with that of *A. thaliana *[15-17]. The transcriptional response of plants to P stress can provide insights into nutrient signal pathways [18] and can inform efforts to improve fertiliser-use efficiency of crop production systems through breeding and nutritional diagnostics [16].

## Results and Discussion

### Genomic-DNA hybridisation and probe-selection

We used *A. thaliana *ATH1-121501 GeneChip^® ^arrays to study the transcriptome of *B. oleracea*. Sequence polymorphisms between the two species are likely to result in an underestimate of transcript abundance if all probes are used within individual probe-sets [13]. Therefore, we selected subsets of probe-pairs from each probe-set on the *A. thaliana *GeneChip^® ^array based on the hybridisation efficiency of genomic DNA from *B. oleracea *to homologous *A. thaliana *GeneChip^® ^array PM probes.

Genomic DNA from *B. oleracea *was biotin-labelled and hybridised to the *A. thaliana *ATH1-121501 GeneChip^® ^array. Probe-sets were selected for subsequent transcriptome analyses if the probe-set was represented by PM probes with gDNA hybridisation intensities above a set threshold. Selection was performed using a .cel file parser script written in the Perl programming language (Xspecies Version 1.1, available at ). The method was optimised empirically by generating 13 probe mask files with gDNA hybridisation intensity thresholds ranging from 0 (i.e. no probe-selection) to 1000. These 13 probe mask files were assessed in turn to investigate the quantification of the transcriptional response of *B. oleracea *to an imposed mineral nutrient (phosphorus; P) stress.

**Figure 1:**
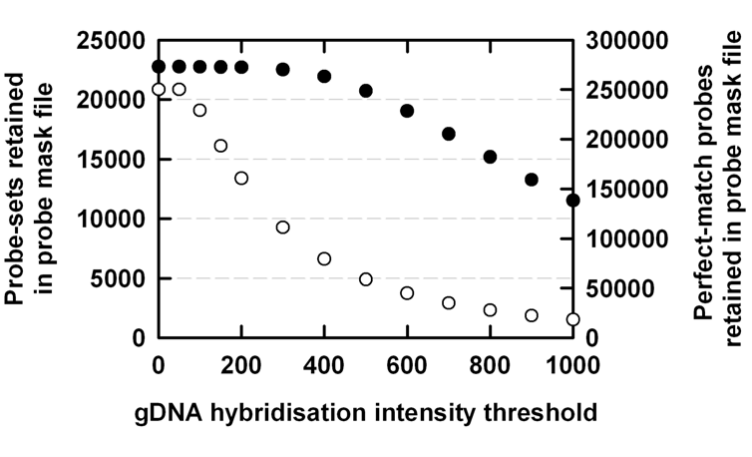
Number of *Arabidopsis thaliana *probe-pairs and probe-sets from the ATH1-121501 GeneChip^® ^array used to study the transcriptome of *Brassica oleracea *var. *alboglabra *cv. A12DHd as a function of the gDNA hybridisation intensity thresholds used to generate the probe mask files. Filled circles are scaled to the left-hand *y*-axis (i.e. probe-sets used in probe mask files) and unfilled circles are scaled to the right-hand *y*-axis (i.e. probe-pairs used in probe mask files). Data were obtained by hybridising genomic DNA from *B. oleracea *to the *A. thaliana *ATH1-121501 GeneChip^® ^array.

*Arabidopsis thaliana *PM probes hybridised extensively to the *B. oleracea *genomic DNA (Figure 1). When the gDNA hybridisation intensity threshold was increased from 0 to 1000 during probe mask file generation, PM probe retention in the probe mask files decreased rapidly. However, the retention of whole probe-sets, representing transcripts, were less sensitive to increases in gDNA hybridisation intensities during probe mask file generation, since only a minimum of one PM probe was required to retain a probe-set. For example, although the probe mask file generated using a gDNA hybridisation intensity threshold of 300 masked > 50 % of all PM probes, 98.8 % of available *A. thaliana *probe-sets were retained.

### Testing DNA probe-selection: transcript abundance in control *Brassica oleracea*

*Brassica oleracea *were grown hydroponically using physiological techniques described elsewhere [15]. Control plants were supplied with a full nutrient solution throughout the experiment; treated (P-starved) plants were supplied with a nutrient solution containing no P for 100 h. Transcriptional responses of *B. oleracea *to phosphate stress were determined by challenging *A. thaliana *ATH1-121501 GeneChip^® ^arrays with total RNA extracted from control and treated plants. Following scanning of the GeneChip^® ^arrays, raw cell intensity data files (.cel files) were loaded into GeneSpring (Silicon Genetics, CA, USA) using 13 probe mask files. For each of the 13 probe-selection conditions, data were prenormalised using Robust Multichip Average (RMA) algorithms [19]. Subsequently, data from the eight GeneChip^® ^arrays (four control and four P-starved samples) were treated as 13 individual 'experimental scenarios' to evaluate probe-selection stringency. Within each 'scenario', data were further standardised within GeneSpring; the signal value from each replicate P-starved plant was standardised to its corresponding control sample to give an expression ratio for each gene.

**Figure 2:**
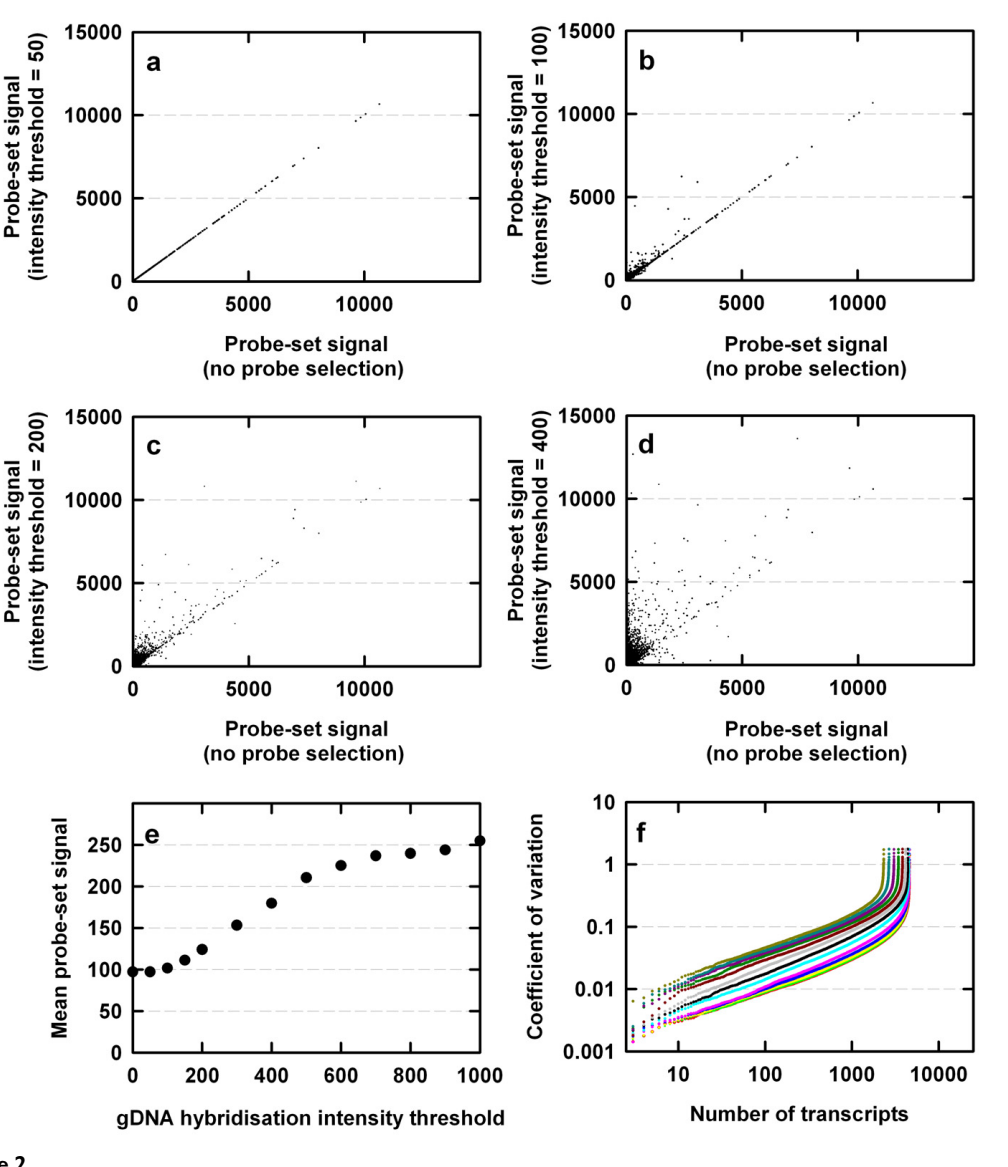
(a) – (d) Probe-set signals of genes in control (P-replete) *Brassica oleracea *var. *alboglabra *cv. A12DHd estimated following probe-selection compared to probe-set signals estimated without probe-selection. Data are presented using probe mask files generated with gDNA hybridisation intensity thresholds of 50, 100, 200 and 400 respectively ((a) – (d)). Mean values (e) and ranked coefficient of variation (f) of probe-set signals of control (P-replete) *B. oleracea *as a function of the gDNA hybridisation intensity thresholds used to generate probe mask files for the transcriptome analysis. In (f), the gDNA hybridisation intensity threshold used to generate probe mask files is indicated by different coloured lines: red (gDNA hybridisation intensity threshold = 0), green (50), yellow (100), blue (150), pink (200), cyan (300), black (400), grey (500), dark red (600), dark green (700), dark pink (800), dark cyan (900), dark yellow (1000). In all panels, total RNA samples were extracted from the shoots of hydroponically-grown control (P-replete) *B. oleracea *(n = 4).

The use of probe masks increased estimates of transcript abundance in *B. oleracea *in both control and P-starved samples (data shown for control samples; Figure 2a–e). The probe mask file generated with a gDNA hybridisation intensity threshold of 50 did not affect estimates of transcript abundance; only nine of 250,206 available PM probes were excluded from this probe mask file. Thus, all probe-sets were retained for subsequent transcriptome analysis (Figures 1, 2a). However, probe mask files generated with gDNA hybridisation intensity thresholds of 100 and above increased estimates of transcript abundance compared to using no probe-selection (Figure 2b–d). Estimates of transcript abundance in *B. oleracea *increased by over 2.5-fold when probe masks were used (Figure 2e). Increasing the gDNA hybridisation intensity threshold during probe mask file generation also increased the coefficient of variation (CV) calculated for ranked transcripts (probe-sets) across the four replicate control arrays (Figure 2f). Thus, if a stringent probe-selection criterion is to be used to analyse experimental transcriptome data, it may be appropriate to increase biological replication. However, the CV of control arrays were minimally affected using probe mask files generated at gDNA hybridisation intensity thresholds below 500 (Figure 2f).

### Testing DNA probe-selection: gene regulation under P stress in *Brassica oleracea*

**Figure 3:**
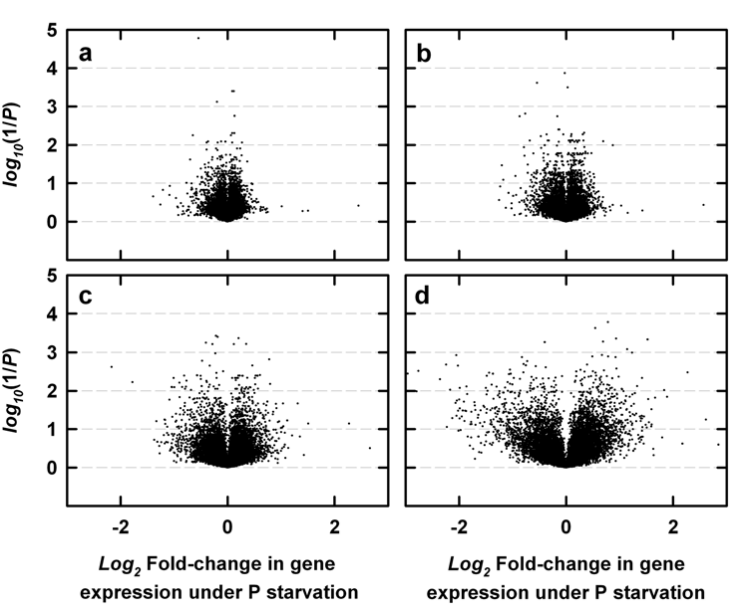
'Volcano' plots illustrating the *log*_2 _of the fold-changes (i.e. the ratio of means for each gene) and inverse significance (i.e. *log*_10 _of the reciprocal of the Benjamini and Hochberg False Discovery Rate multiple test corrected *P*-value derived from a one-way ANOVA with the Benjamini and Hochberg FDR multiple testing correction) in gene expression differences between control and P-starved *Brassica oleracea *var. *alboglabra *cv. A12DHd. Total RNA samples were extracted from control *B. oleracea *shoots and from the shoots of plants grown in the absence of P for 100 h (n = 4). (a) no probe-selection used during transcriptome analysis, (b), (c), (d) using probe mask files during transcriptome analysis, generated at gDNA hybridisation intensity thresholds of 200, 400 and 1000 respectively. Data in Additional file 1.

**Figure 4:**
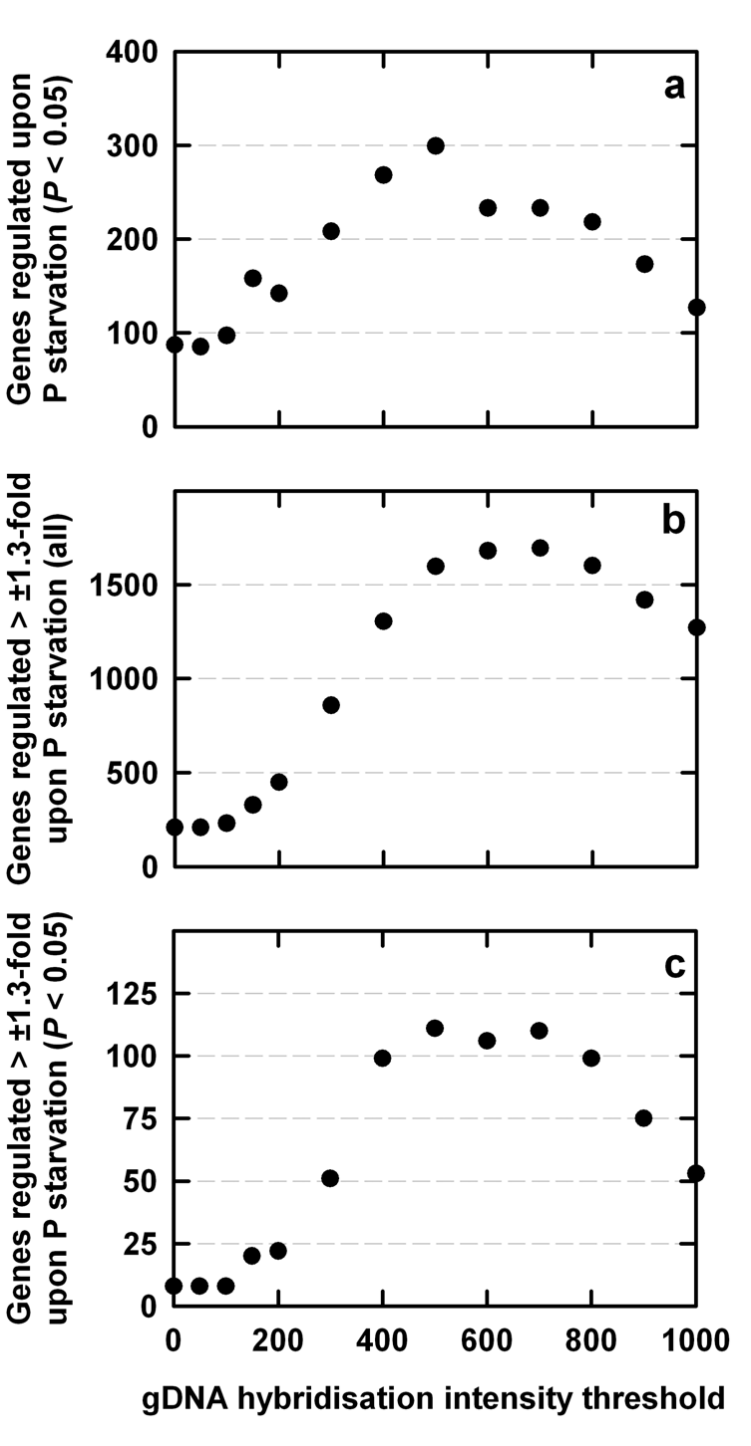
Gene regulation under P-starvation in *Brassica oleracea *var. *alboglabra *cv. A12DHd as a function of the gDNA hybridisation intensity threshold used to generate probe mask files for the transcriptome analysis. Total RNA samples were extracted from control *B. oleracea *shoots and from the shoots of plants grown in the absence of P for 100 h (n = 4). (a) genes significantly regulated under P starvation at Benjamini and Hochberg False Discovery Rate multiple test corrected (BH-FDR MTC) *P *< 0.05. (b) genes regulated > ± 1.3-fold under P starvation. (c) genes significantly regulated > ± 1.3-fold under P starvation (BH-FDR MTC *P *< 0.05). Data in Additional file 1.

Gene expression ratios were log-normally distributed when experiments were analysed using any of the 13 probe mask files (data not shown). For each experimental interpretation, fold-change differences in gene expression were calculated as the ratio of normalised means for each gene in the treated sample relative to the control sample. A one-way ANOVA, with a Benjamini and Hochberg False Discovery Rate (BH-FDR = 0.05) multiple testing correction (MTC) applied, was used to test if transcript abundance was significantly different between control and P-starved samples. Increasing the gDNA hybridisation intensity threshold from 0 to 500 for probe mask file generation increased the sensitivity of the ATH1-121501 GeneChip^® ^array to detect regulation of gene expression in *B. oleracea *under P stress, in terms of significant > ± 1.3-fold differences in gene expression between P-starved and control samples (Figures 3, 4).

Estimates of fold-differences in gene expression (P-starved samples *versus *control samples) increased using probe mask files generated with gDNA hybridisation intensity thresholds up to 500 (Figure 3, 4c). 'Volcano' plots summarise these effects (Figure 3a–c). In volcano plots, the *y*-axis represents the *log*_10 _of the reciprocal of the one-way ANOVA *P-*value corrected for multiple testing using the BH-FDR; the *x-*axis represents the *log*_2 _of the fold-change in gene expression. There was a 13-fold increase in the number of genes identified as differentially regulated > ± 1.3-fold under P stress using a probe mask file generated with a gDNA hybridisation intensity threshold of 500 (Figures 3, 4c). The use of probe mask files generated with higher gDNA hybridisation intensity thresholds (up to 1000) resulted in a substantial loss of probe-sets available for transcriptome analysis (Figures 1, 3d). However, the number of genes identified as differentially regulated > ± 1.3-fold was still much greater when using the probe mask file generated with a gDNA hybridisation intensity threshold of 1000 than when probe-selection was not used, even though only 7.3 % of the total number of PM probes on the GeneChip^® ^array were used (Figure 4c). Estimates of the number of genes significantly differentially regulated under P stress (at BH-FDR MTC *P *< 0.05) increased when probe mask files were used (Figure 4a). For example, using a probe mask file generated with a gDNA hybridisation intensity threshold of 500, 111 genes were estimated as significantly differentially regulated, compared to only eight genes regulated using no probe-selection.

An optimal probe-selection strategy for transcriptional analysis of *B. oleracea *is to use a probe mask file generated at a gDNA hybridisation intensity threshold of 200 or greater. Although estimates of fold-differences in gene expression in P-starved *versus *control samples were greatest using probe mask files generated with gDNA hybridisation intensity thresholds of up to 500 (Figure 4c,d), there was a significant loss of available probe-sets for transcriptome analysis with gDNA hybridisation intensity thresholds > 500. Further, estimates of the number of significantly regulated genes declined using probe mask files generated at DNA hybridisation intensity thresholds > 500.

### Biological significance of genes regulated under P stress in *Brassica oleracea*

Following probe-selection using a gDNA hybridisation intensity threshold of 400, 99 genes were identified as significantly differentially regulated in the shoots of *B. oleracea *following the withdrawal of P from the nutrient solution (BH-FDR MTC *P *< 0.05; Additional file 1). Of these genes, 39 had higher transcript abundance in P-starved samples than in control samples and 60 had lower transcript abundance in P-starved samples than in control samples. Data for all genes, analysed with all probe mask files generated at gDNA hybridisation intensity thresholds from 0 to 1000, are presented in Supplementary Table I.

Sequence polymorphisms between *A. thaliana *and *B. oleracea *are likely to result in the identification of homologous genes or alternative members of gene families in addition to possible gene orthologues. For this reason, we compared previously published studies on the response of *A. thaliana *to P starvation [15-17] at the level of individual gene and also at the level of the gene family and functional category. Several homologous genes responded similarly to P starvation in *B. oleracea *and *A. thaliana*. For example, *SQD2 *(At5g01220), which is involved in sulpholipid biosynthesis, increases its expression in responses to P starvation in *A. thaliana *[15-17,20-22]. A homologue of this gene had higher hybridisation signals in P-starved *B. oleracea *than in control samples. The signal values for *SQD2*, obtained using the probe mask file generated with a gDNA hybridisation intensity threshold of 400, were based on two probes, in contrast to 11 probes when no probe-selection was used. The use of probe-selection increased the significance and differences in transcript abundance between P-starved and control plants (*SQD2*, 1.60 ± 0.2, BH-FDR MTC *P *= 0.047; mean normalised signal ratio ± 1 S.D.) than in the absence of probe-selection (*SQD2*, 1.53 ± 0.58, BH-FDR MTC *P *= 0.382).

Ribonucleases, phosphatases and phosphodiesterases are involved in the recycling of P in plants during P starvation [16,23,24]. The hybridisation intensity of the ribonuclease *RNS2 *(At2g39780) was higher in P-starved samples compared to control samples, and is thought to be involved in the release of P from internal sources during P starvation [23]. The signal value for *RNS2*, obtained using the probe mask file generated with a gDNA hybridisation intensity threshold of 400, was based on six probes in contrast to 11 probes when no probe-selection was used. The use of probe-selection increased normalised signal ratios in P-starved plants compared to control plants to a greater amount (*RNS2*, 1.92 ± 0.43, BH-FDR MTC *P *= 0.06, mean normalised signal ratio ± 1 S.D.) than in the absence of probe-selection, where the up-regulation of the gene was not detected (*RNS2*, 1.07 ± 0.06, BH-FDR MTC *P *= 0.43).

Higher hybridisation signals in P-starved plants compared to control samples were also observed for genes belonging to gene families or functional groups which have previously been observed responding to P starvation in *A. thaliana*, although not significantly [15-17]. These include genes involved in the transport of phosphate and phosphate-containing compounds and carbohydrates, indicating a change in the internal economy of P use in response to P starvation (Additional file 1). For example, at a gDNA hybridisation intensity of 400, two sucrose-phosphate synthases, and a malic enzyme had higher hybridisation intensities for in P-starved compared to control samples, whilst lower hybridisation intensities for genes involved in alternative glycolosis reactions that conserve P during P starvation conditions [16] were noted. There are other notable similarities in the P starvation response of *B. oleracea *with published responses of *A. thaliana *to P starvation at the level of the gene family. These include transcription factors from the zinc finger, F-box, bHLH and myb families, genes involved in flavanoid biosynthesis and genes encoding proteins from the cytochrome P450, glycosyl hydrolase, and peroxidase families.

## Conclusion

Since GeneChip^® ^arrays are available for only a small number of species, we have developed a novel probe-selection method to study the transcriptome of a plant or animal species for which GeneChip^® ^arrays are not available. Genomic DNA from *B. oleracea *was labelled and hybridised to the *A. thaliana *ATH1-121501 GeneChip^® ^array. Perfect-match *A. thaliana *probes which hybridised to the *B. oleracea *genomic DNA above selected hybridisation intensities were selected for subsequent *B. oleracea *transcriptome analysis using probe mask files generated using a .cel file parser script. Software to create probe mask files is freely available  and is designed to facilitate the analysis of the transcriptomes of a wide range of other species in the absence of custom arrays.

Probe-selection was tested by quantifying the transcriptional response of *B. oleracea *to a mineral nutrient (phosphorus; P) stress using probe mask files generated at gDNA hybridisation intensity thresholds ranging from 0 (i.e. no probe-selection) to 1000. A probe mask file generated at a gDNA hybridisation intensity threshold of 400 masked > 68 % of all of the *A. thaliana *probe-pairs from the subsequent transcriptome analysis whilst retaining 96.4 % of the total available probe-sets. Increasing the gDNA hybridisation intensity thresholds for probe mask file generation increased the sensitivity of the GeneChip^® ^array to detect regulation of gene expression in *B. oleracea *under P stress by up to 13-fold. Ninety-nine genes were significantly regulated in the shoots of *B. oleracea *under P stress (BH-FDR MTC *P *< 0.05). Confirmatory analyses using quantitative PCR and reporter-genes fused to nutrient responsive promoters have already been conducted for several P stress regulated genes in *A. thaliana *[15,16] and detailed confirmatory and functional molecular analysis of these genes is now clearly required in *B. oleracea *to resolve the biological significance of the P stress response. Understanding P stress responses in plants is likely, (i) to hasten the development of more nutrient efficient varieties of crops, (ii) to improve our understanding of nutrient signalling pathways, and (iii) to yield rapid advances in nutritional diagnostics, in particular if unit costs for microarray analyses continue to decrease [16,18]. We appreciate that the resolution and clarity of the probe mask method will be influenced by gene duplication and will necessitate validation of the potential homologues found in the target species, however this caution is generally true in the design of any new microarray. Most array users are primarily interested in transcript differences between samples as clues to further investigation of a gene or gene family. This technique enables such differential expression indicators for genes from unusual species and additionally provides putative model organism gene homologue ontologies via the standard Affymetrix gene annotation. As a pragmatic method based on gDNA hybridisation the technique requires investigation of a range of thresholds to systematically test probe-selection at the bioinformatics level. Re-optimisation of gDNA hybridisation thresholds is particularly important for new species given that gDNA quality and origin may affect overall absolute hybridisation intensities per experiment but should not affect relative ranking of intensities between hybridisations of the same genome. The technique therefore allows an economical analysis of any RNA hybridisations performed, since gDNA-based probe-selection can be optimised at any time without repeating the RNA work. The method also requires minimal setup outlay represented by one representative gDNA hybridisation and some straightforward bioinformatics time, in contrast to the costs of manufacturing even a simple single oligo array. More importantly, however, gDNA-based probe-selection could facilitate transcriptional profiling of a wide range of plant and animal species of agricultural, ecological and evolutionary importance, even in the absence of available genomic information.

## Materials and methods

### Genomic DNA hybridisation and probe-selection

Probe-pairs from the *A. thaliana *ATH1-121501 GeneChip^® ^array (Affymetrix, Santa Clara, CA, USA) were selected for transcriptome analysis of *B. oleracea *using a gDNA-based probe-selection strategy based on the hybridisation of gDNA to the PM probe. Total genomic DNA was extracted from 5 g of *B. oleracea *var. *alboglabra *cv. A12DHd leaf tissue using a DNeasy Plant mini kit (Qiagen Ltd, Crawley, UK). Genomic DNA was labelled using the Bioprime DNA labelling System (Invitrogen, Paisley, UK) and subsequently hybridised to Affymetrix ATH1-121501 GeneChip^® ^arrays for 16 h at 45°C using standard Affymetrix hybridisation protocols. Subsequently, the GeneChip^® ^array was scanned on an Affymetrix G2500A GeneArray scanner and a cell intensity file (.cel file) was generated using Microarray Analysis Suite (MAS Version 5.0; Affymetrix). This .cel file contained the gDNA hybridisation intensities between *B. oleracea *genomic DNA fragments and all *A. thaliana *probes. Probe-pairs from the .cel file were selected for subsequent transcriptome analysis using a .cel file parser script (Xspecies Version 1.1) written in the Perl programming language . The Perl script was designed to create probe mask (.cdf) files compatible with a range of microarray analysis software packages. A probe-set was selected when it was represented by one or more PM probe-pair(s) per probe-set (i.e. a minimum of 25 bp identical probe sequence to *A. thaliana *was required for subsequent transcriptome analysis of *B. oleracea*). There was no *a priori *restriction of a suitable gDNA hybridisation intensity threshold for probe mask file generation in a target species. Thus, the algorithm was designed to allow a user-specified gDNA hybridisation intensity threshold for probe mask file generation to be set. Files (cdf.) were therefore generated using a range of gDNA hybridisation intensity thresholds (from 0 to 1000). The Perl algorithm and *B. oleracea *DNA .cel files are freely available from  along with similar files for other species. The Perl masking script and instructions for using the script are also available as Additional file 2 and Additional file 3.

### Testing the gDNA-based probe-selection strategy: determining the transcriptional response of *B. oleracea *to phosphate stress

*Brassica oleracea *var. *alboglabra *cv. A12DHd were grown hydroponically from seed in a system described in Hammond *et al.*, [15]. Control plants were supplied with nutrient solution containing all nutrients throughout the experiment whilst treated plants were supplied with a nutrient solution containing no P for 100 h. Replicate samples (each replicate comprising 8–10 individual plants) were harvested from control and treated plants, mid way through the photo-period, and snap frozen in liquid nitrogen. All plants had approximately 6–8 leaves at harvest. Three biological replicates and one technical replicate were used for transcriptome analysis.

Tissue samples, previously stored at -70°C, were placed in liquid nitrogen before grinding. To each sample, 1 ml of TRIzol reagent (Invitrogen) was added and total RNA was subsequently extracted as described previously [15]. Total RNA yield and purity were determined using an Agilent 2100 Bioanalyser (Agilent Technologies, Stockport, Cheshire, UK). Approximately 5 μg of total RNA was reverse transcribed at 42°C for 1 h to generate first strand cDNA using 100 pmol oligo dT(24) primer containing a 5'-T7 RNA polymerase promoter sequence, 50 mM Tris-HCl (pH 8.3), 75 mM KCl, 3 mM MgCl_2_, 10 mM dithiothreitol (DTT), 10 mM dNTPs and 200 units SuperScript II reverse transcriptase (Invitrogen Life Technologies). Following first strand cDNA synthesis, second strand cDNA was synthesised using 10 units of *Escherichia coli *polymerase I, 10 units of *E. coli *DNA ligase and 2 units of RNase H in a reaction containing 25 mM Tris-HCl (pH 7.5), 100 mM KCl, 5 mM MgCl_2_, 10 mM (NH_4_)SO_4_, 0.15 mM b-NAD^+ ^and 10 mM dNTPs. The second strand synthesis reaction proceeded at 16°C for 2 h before 10 units of T4 DNA polymerase was added and the reaction allowed to proceeded for a further 5 minutes. The reaction was terminated by adding 0.5 M EDTA. Double stranded cDNA products were purified using the GeneChip^® ^Sample Cleanup Module (Affymetrix). The synthesised cDNAs were *in-vitro *transcribed by T7 RNA polymerase (Enzo BioArray High Yield RNA Transcript Labelling Kit, Enzo Life Sciences Inc., Farmingdale, NY, USA) using biotinylated nucleotides to generate biotinylated complementary RNAs (cRNAs). The cRNAs were purified using the GeneChip^® ^Sample Cleanup Module (Affymetrix). The cRNAs were then randomly fragmented at 94°C for 35 minutes in a buffer containing 40 mM Tris-acetate (pH 8.1), 100 mM potassium acetate, and 30 mM magnesium acetate to generate molecules of approximately 35 to 200 bp. Affymetrix *A. thaliana *ATH1-121501 GeneChip^® ^arrays were hybridised with 15 μg of fragmented labelled cRNA for 16 h at 45°C as described in the Affymetrix Technical Analysis Manual. GeneChip^® ^arrays were stained with Streptavidin-Phycoerythrin solution and scanned with an Affymetrix G2500A GeneArray scanner.

Microarray Analysis Suite (MAS Version 5.0; Affymetrix) was used to generate .cel files for each of the biological RNA replicates by scanning and computing summary intensities for each probe without the use of probe mask files. These .cel files were loaded into GeneSpring (Agilent Technologies) analysis software package using the Robust Multichip Average (RMA) pre-normalisation algorithm (Irizarry *et al.*, 2003). During .cel file loading and pre-normalisation, .cel files were interpreted using either, (1) the *A. thaliana *.cdf file (i.e. with no probe-selection used), or (2) using .cdf files generated from the gDNA .cel file with gDNA hybridisation intensity thresholds from 50 to 1000. Following RMA pre-normalisation and masking of individual probes, further standardisations were applied to the probe-set raw signal value in GeneSpring. For each replicate array, each probe-set signal value from treated (P-starved) samples was standardised to the probe-set signal value of its corresponding control sample to give gene expression ratios between the two conditions. Genes with differential hybridisation intensities between P-starved and control samples were identified using a one-way ANOVA, with a Benjamini and Hochberg false discovery rate (0.05) multiple testing correction applied, to identify genes that were significantly differentially expressed between the two conditions. Genes with Benjamini and Hochberg FDR multiple test corrected *P*-values less than 0.05 were considered to be differentially regulated under P starvation.

## Supplementary Material

Additional File 1 An archive of the mean probe-set signals of all genes in P-starved versus control (P-replete) *Brassica oleracea *var. alboglabra cv. A12DHd. Raw data were analysed with the RMA pre-normalisation algorithm using the ATH1-121501 probe mask file (no probe selection) or a custom probe mask file. The custom probe mask files were generated by hybridising gDNA from *B. oleracea *to Affymetrix *A. thaliana *ATH-121501 GeneChip^® ^arrays and selecting probe-pairs in which *B. oleracea *gDNA hybridisation intensity values were greater than 50, 100, 150, 200, 300,...1000. Signal intensities are estimates from total RNA extracted from the shoots of hydroponically-grown plants (n = 4) under P-deficient (raw) and P-replete (control) conditions. The *P*-values are derived from a one-way ANOVA using a Benjamini and Hochberg False Discovery Rate (0.05) multiple testing correction. Click here for file

Additional File 2 An archive containing all of the Perl scripts necessary in order to produce new CDF files for use in analysing cross-species hybridisation results. Click here for file

Additional File 3 (CDF_filtering script instructions.doc : DOC file). An instruction document containing details for using the scripts contained within Additional file 2. Click here for file
