# Supplementary material for: Using genomic DNA-based probe-selection to improve the sensitivity of high-density oligonucleotide arrays when applied to heterologous species
Source: Plant Methods. 2005 Nov 9;1:10. doi: 10.1186/1746-4811-1-10 (PMC1308859; doi:10.1186/1746-4811-1-10)
Supplement: Additional File 3 — (CDF_filtering script instructions.doc : DOC file). An instruction document containing details for using the scripts contained within Additional file 2. [file 1746-4811-1-10-S3.doc]

CDF Xspecies filtering perl scripts v1.1 - instructions for use:

1. You will need perl installed

(e.g. activeperl for Windows - native for other OS eg. OSX, LINUX)

2. Unzip the CDF_masking.zip archive to your chosen location.

3. Acquire a DNA-hybridisation CEL file and an original Affymetrix CDF file for the target chip used, put them in the CDF_masking folder

(e.g. for arabidopsis ATH1 chip use the ATH1-121501.CDF from Affymetrix.com)

4. Run easy_script.pl and follow the instructions.

If the script runs slowly, please ensure that you are using a machine with more than 512MB memory (recommended).
